# Regional-Scale Estimation of Maize Plant Moisture Content in Arid Regions Integrating Multi-Source Remote Sensing and Machine Learning

**DOI:** 10.3390/plants15132044

**Published:** 2026-07-01

**Authors:** Jixuan Yan, Xuchun Li, Zichen Guo, Wenning Wang, Qiang Li, Zhuo Che, Guang Li, Weiwei Ma, Yinshan Ma, Kejing Cheng, Jiaqin Yuan

**Affiliations:** 1 State Key Laboratory of Aridland Crop Science, College of Water Conservancy and Hydropower Engineering, Gansu Agricultural University, Lanzhou 730070, China; 2 Gansu Provincial Agricultural Smart Water-Saving Technology Innovation Center, Lanzhou 730070, China; 3 Gansu Provincial Agricultural Construction Project Management Station, Lanzhou 730070, China; 4 College of Agriculture and Ecological Engineering, Hexi University, Zhangye 734000, China; 5 College of Forestry, Gansu Agricultural University, Lanzhou 730070, China

**Keywords:** silage maize, cross-scale calibration, feature selection, precision irrigation, arid agriculture

## Abstract

Agricultural production in arid regions is strongly constrained by water stress, making timely evaluation of crop water conditions increasingly important. However, conventional measurements of plant moisture content (PMC) primarily rely on destructive oven-drying methods, which are not only labor-intensive and time-consuming but also constrained by limited sample size and spatial coverage. These shortcomings make it difficult to capture the spatial heterogeneity of crop water status across large agricultural regions, thereby restricting regional-scale water diagnosis and precision irrigation decision-making. Focusing on silage maize cultivated in the arid region of Gansu Province, China, this work develops a regional PMC estimation approach by combining multi-source remote sensing data. High-resolution unmanned aerial vehicle (UAV) observations were integrated with Sentinel-2 and Sentinel-3 imagery, while radiometric and temperature corrections were applied to improve data consistency. A set of spectral, textural, and thermal features was derived from multispectral, visible, and thermal infrared datasets. Feature selection based on Pearson correlation was then carried out, followed by the construction of three models, namely Random Forest (RF), Support Vector Machine (SVM), and Partial Least Squares Regression (PLSR). Among them, the RF model performed more reliably, achieving a validation R^2^ of 0.92 with relatively low prediction error. In addition, calibration using UAV data led to a clear improvement in satellite-based estimates, with R^2^ increasing from 0.52–0.62 to 0.71–0.74. The generated PMC maps captured both the temporal decline during the growing season and the spatial variability across the study area. Overall, the proposed approach offers a practical option for large-scale monitoring of crop water status and can support irrigation management in water-limited environments.

## 1. Introduction

Climate change is increasingly influencing agricultural systems worldwide, with particularly strong effects observed in arid regions. In such environments, agricultural production is subject to growing uncertainty [1], as drought and heat stress have emerged as major limiting factors for crop growth, influencing plant development, physiological processes, reproductive stages, and ultimately food security [2]. Meanwhile, declining precipitation and altered rainfall patterns have contributed to a noticeable intensification of drought conditions on a global scale. These pressures are even more pronounced in arid and developing regions, where existing issues—including poverty, food insecurity, malnutrition, limited healthcare access, and economic vulnerability—are further aggravated [3]. Under these circumstances, improving the understanding of current agricultural practices and exploring more adaptive management strategies becomes increasingly important. At the same time, conducting crop assessments over large areas remains challenging due to spatial heterogeneity and data limitations [4]. In this context, remote sensing has gradually been adopted as an effective tool for monitoring soil conditions and crop growth, offering a practical alternative to conventional field-based measurements.

Plant moisture content (PMC) is widely regarded as a key indicator of crop water status and is closely linked to yield formation, product quality, and the effectiveness of precision irrigation practices in modern agriculture [5]. Accurate and timely estimation of PMC therefore plays an important role in improving water use efficiency and supporting more sustainable agricultural management, particularly under increasing environmental stress. Although irrigation is essential for maintaining crop growth, issues related to inefficient water use and excessive irrigation remain common, especially in arid regions such as the Hexi Corridor in China [6]. These challenges are further intensified by climate change, which not only increases heat stress but also alters precipitation patterns, making water management more complex. Under such conditions, there is a growing demand for more advanced and reliable monitoring approaches to better cope with water scarcity and enhance system adaptability. Conventional methods for measuring PMC are mainly based on destructive sampling, where plant materials are collected, dried, and weighed to determine their water content [7]. While these approaches can achieve high accuracy, they are labor-intensive and time-consuming, and their applicability is limited when large areas or frequent observations are required. This limitation has prompted increasing interest in developing non-destructive techniques that can improve monitoring efficiency and support sustainable water resource management [8]. Recent developments in remote sensing have shown considerable potential for providing near-real-time information on PMC, making it a promising tool for precision irrigation and climate adaptation.

Although ground-based measurements of PMC can provide detailed and high-resolution information, their spatial coverage is inherently limited. By comparison, remote sensing enables observations over much larger areas, making it more suitable for regional-scale monitoring. Maimaitijiang et al. showed that UAV multispectral data with machine learning enables accurate estimation of moisture content (R^2^ > 0.80) and senescence phenotyping (R^2^ = 0.82–0.88) [9]. Satellite-based approaches, however, are often restricted by relatively long revisit intervals, which reduces their ability to capture rapid temporal changes. In addition, freely available satellite data usually lack the spatial resolution required for precision agriculture, while commercial high-resolution imagery is often costly, limiting its broader application [10]. UAV-based remote sensing offers a flexible alternative, with advantages such as finer spatial resolution and the ability to acquire data in near-real time. Even so, applying UAVs over large areas can be both time-consuming and expensive, which constrains their use in extensive monitoring tasks [11]. Given these limitations, combining ground observations with UAV and satellite data has attracted increasing attention as a potential way to overcome the weaknesses of individual data sources. Despite this, such integrated approaches are still developing, particularly in studies that simultaneously incorporate satellite imagery, UAV data, and ground measurements [12]. Constraints related to spatial resolution, spectral characteristics, and sensor coverage further limit the effectiveness of relying on a single data source for large-scale analysis [13]. In this context, making use of the complementary strengths of multiple remote sensing platforms offers a practical direction for improving PMC estimation.

In recent years, the integration of remote sensing technology and machine learning has opened new avenues for monitoring crop water status. With respect to single data sources, unmanned aerial vehicle (UAV)-based multispectral remote sensing has been widely employed for estimating crop water content. Wang et al. utilized UAV multispectral imagery combined with random forest regression (RFR) to estimate leaf water content in maize, and found that the RFR model performed best at the seedling stage, achieving a relative root mean square error (RRMSE) of 2.99% [10]. Li et al. estimated plant moisture content (PMC) at different phenological stages of silage maize in the Hexi region using UAV multispectral data and ensemble learning methods [14]. In the domain of water stress diagnosis, researchers have constructed indicators such as the crop water stress index (CWSI) based on UAV thermal infrared and multispectral data, and combined them with machine learning approaches to effectively monitor the water status of dryland crops including maize and sorghum [11]. With the growing demand for broader spatial coverage, multi-source remote sensing data fusion has increasingly become a research hotspot. Liu et al. proposed a fusion method for UAV multispectral and Sentinel-2 satellite imagery based on additive wavelet transform (AWT), and combined it with five machine learning models to estimate fuel moisture content (FMC), equivalent water thickness (EWT), and canopy water content (CWC) in mango canopies. Their results demonstrated that the fusion of Sentinel-2 data significantly improved the estimation accuracy for all parameters. In another study, the fusion of UAV and Sentinel-2 data, coupled with the PROSAIL-5D radiative transfer model and a genetic algorithm-optimized BP neural network, was used to estimate fuel moisture content in agroecosystems, achieving an R^2^ of 0.765 [8]. For soil water content estimation, Li et al. integrated thermal infrared, multispectral, and meteorological data to construct a multidimensional remote sensing feature set comprising CWSI, NDVI, TVDI, and TDDI, and systematically evaluated the performance of four machine learning algorithms—SVR, RFR, GBDT, and CatBoost—in estimating soil water content in cotton fields. Furthermore, the combination of multimodal UAV data and machine learning has been shown to enable high-accuracy PMC monitoring, providing robust support for data-driven precision irrigation scheduling. Despite these significant advances, several outstanding issues remain to be addressed. First, most existing studies are confined to a single data source (UAV or satellite) or a single spatial scale, and lack a regional-scale application framework that systematically calibrates and integrates UAV high-spatial-resolution data with satellite wide-coverage data. Second, few studies have simultaneously incorporated multidimensional information—such as spectral indices, textural features, and thermal characteristics—and systematically analyzed the contributions of each feature across different growth stages. Finally, regional-scale estimation of PMC for silage maize, a specific crop system in arid regions, remains relatively understudied.

To address the aforementioned limitations, the objectives of this study are fourfold: (1) to propose a cross-scale calibration framework for multi-source remote sensing data from UAV and satellite platforms, so as to resolve systematic inconsistencies among the different data sources; (2) to construct a multi-dimensional feature set that integrates 10 spectral indices, 8 texture-based composite indicators, and 4 temperature-related variables, and to systematically select the optimal feature combinations for each growth stage via Pearson correlation analysis; (3) to systematically compare the performance of three models—random forest (RF), support vector machine (SVM), and partial least squares regression (PLSR)—in estimating plant moisture content (PMC); and (4) to generate PMC spatial mapping from the UAV plot scale to the satellite regional scale, thereby providing an operational technical framework for precision irrigation management in arid regions.

## 2. Results

### 2.1. Variation in Plant Moisture Content During Maize Growth

**Figure 1:**
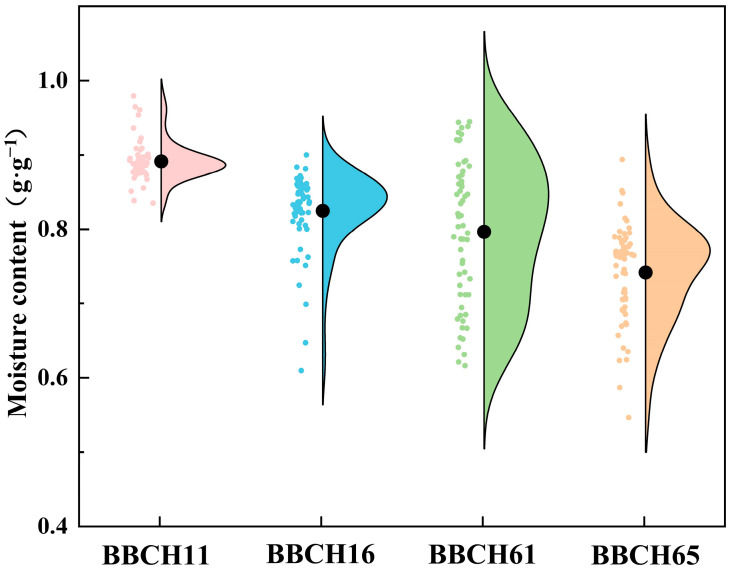
Variation in plant moisture content across different growth stages in 2025.

Variations in PMC were observed across different growth stages, reflecting the combined influence of environmental conditions and plant physiological status. In this study, samples were collected at four key growth stages, namely BBCH 11, BBCH 16, BBCH 61, and BBCH 65, with 60 samples obtained at each stage. The descriptive statistics of PMC for each stage are summarized in Table 1. The statistical results show noticeable variability in PMC across all samples and within individual growth stages, as indicated by the range, standard deviation, and coefficient of variation. As shown in Figure 1, PMC generally decreased as the growth period progressed. In addition, the distribution of PMC values approximated normality, with no evident outliers observed.

### 2.2. Feature Variable Selection

**Figure 2:**
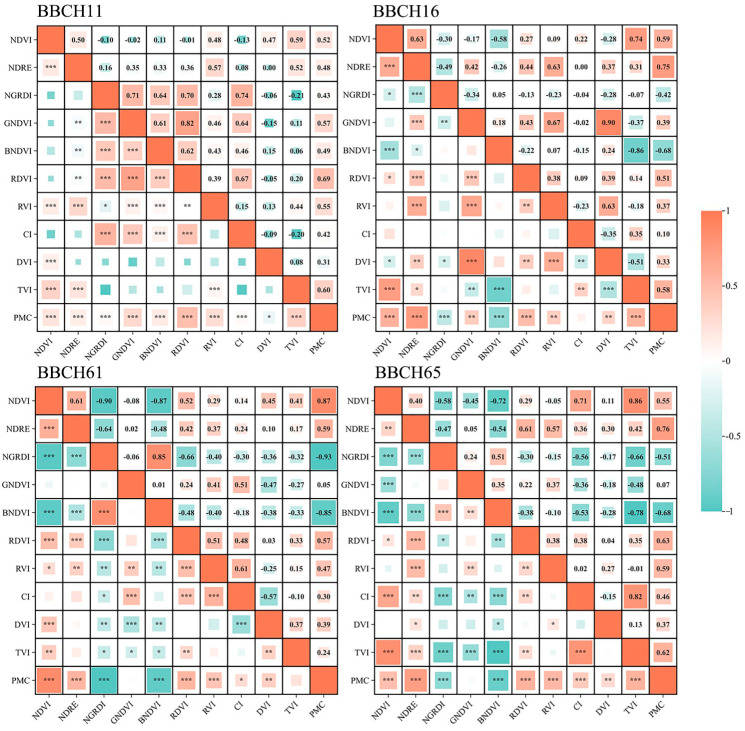
Relationships between PMC and vegetation indices in silage maize. Significance levels: *** *p* < 0.001, ** *p* < 0.01, * *p* < 0.05.

**Figure 3:**
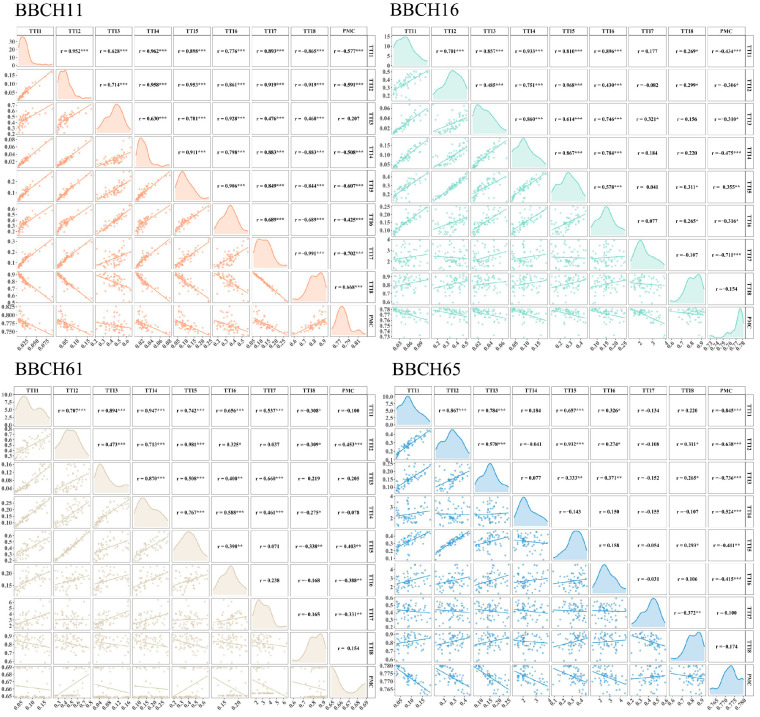
Relationships between PMC and texture features in silage maize. Significance levels: *** *p* < 0.001, ** *p* < 0.01, * *p* < 0.05.

**Figure 4:**
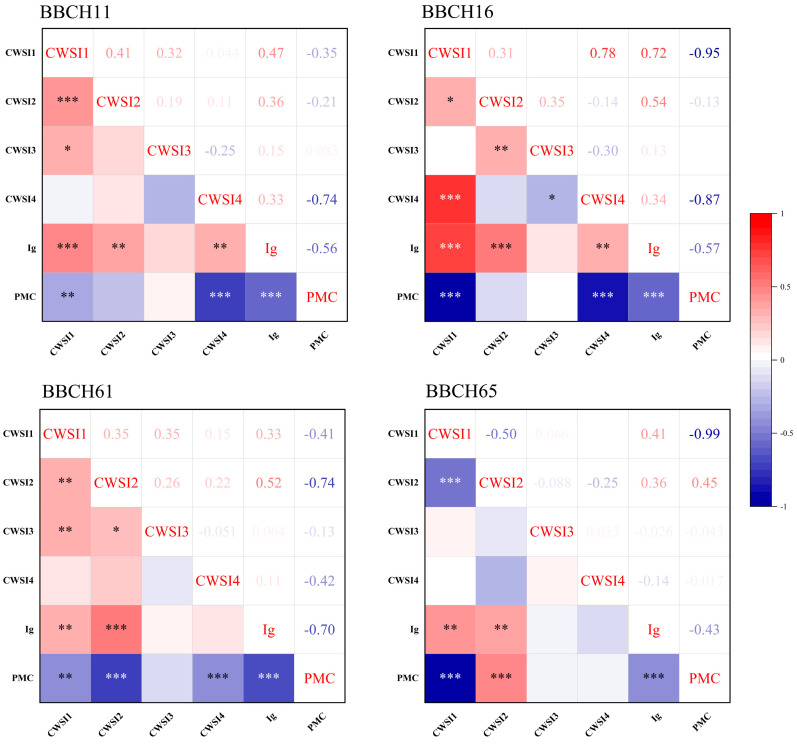
Relationships between PMC and temperature-related features in silage maize. Note: *, **, and *** denote significance at *p* < 0.05, *p* < 0.01, and *p* < 0.001, respectively.

Pearson correlation analysis was conducted to examine the relationships between UAV-derived multispectral vegetation indices, texture features, temperature features, and plant moisture content. The analysis aimed to identify effective feature variables for PMC estimation at different growth stages. The corresponding results are shown in Figure 2, Figure 3 and Figure 4. The correlations between feature variables and PMC varied noticeably across growth stages. Most variables exhibited statistically significant relationships with PMC, with several showing strong significance (*p* < 0.001).

At the vegetation index level, several indices, including NDVI, NDRE, GNDVI, RDVI, and RVI, showed moderate to strong correlations with PMC across multiple growth stages (|r| > 0.4), with positive relationships being dominant. Among them, NDVI (r = 0.87) and NGRDI (r = −0.93) at BBCH 61 exhibited the strongest correlations. In addition, NDVI and NDRE remained significantly correlated with PMC throughout all growth stages, highlighting their importance as spectral indicators. For texture features, TTI1, TTI2, TTI5, and TTI7 were strongly correlated with PMC, particularly at BBCH 11 and BBCH 65 (|r| > 0.4), with negative correlations prevailing. This suggests that lower PMC is associated with increased canopy texture heterogeneity. Notably, TTI7 at BBCH 16 (r = −0.711) and TTI1 at BBCH 65 (r = −0.845) showed relatively strong relationships. Regarding temperature features, CWSI1 exhibited very strong negative correlations with PMC at BBCH 16 (r = −0.95) and BBCH 65 (r = −0.99) stages. Meanwhile, CWSI4 and Ig also showed significant correlations across multiple stages, indicating the sensitivity of water stress-related indices to changes in plant water status. Based on a threshold of |r| > 0.4, representative variables were selected from each feature category for different growth stages, providing inputs for subsequent PMC estimation models.

### 2.3. PMC Inversion Models Based on Feature Selection

**Figure 5:**
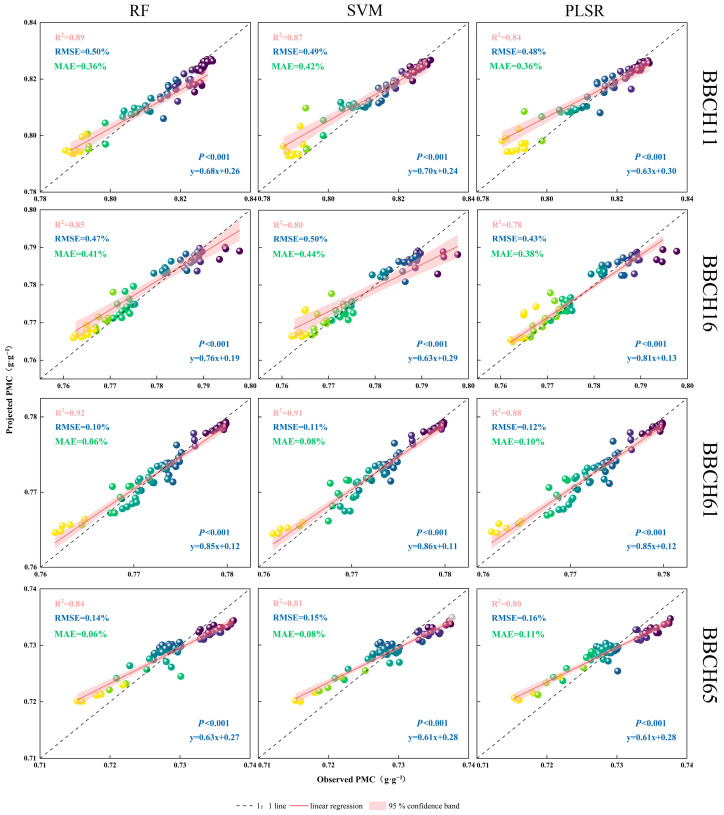
Performance of PMC models across different growth stages.

Using UAV-derived features and the selected optimal variable combinations, PMC estimation models were developed based on Random Forest (RF), Support Vector Machine (SVM), and Partial Least Squares Regression (PLSR). The dataset was split into training and testing subsets at a ratio of 2:1. Model performance across different growth stages is presented in Figure 5.

Overall, the RF model achieved the best performance among the three approaches, showing consistently higher R^2^ values and lower RMSE across all growth stages. The highest accuracy was obtained at BBCH 61 (R^2^ = 0.92, RMSE = 0.10%, MAE = 0.06%), followed by BBCH 11 (R^2^ = 0.89, RMSE = 0.50%, MAE = 0.36%). BBCH 16 and BBCH 65 showed slightly lower accuracy, with R^2^ values of 0.85 and 0.84, respectively. In comparison, the SVM and PLSR models exhibited relatively lower predictive performance, with R^2^ values ranging from 0.80 to 0.91 and 0.78 to 0.88, respectively. Despite these differences, all models showed statistically significant relationships (*p* < 0.001), indicating reliable predictive capability. The regression slopes ranged from 0.61 to 0.86, with intercepts between 0.11 and 0.30, suggesting variations in model sensitivity across growth stages. BBCH 61 showed the closest agreement between predicted and observed values, as indicated by its regression characteristics. The RF model produced predictions that were more closely aligned with the 1:1 line and exhibited a narrower confidence interval, indicating higher stability and accuracy. Based on these results, RF was identified as the most suitable model for PMC estimation across different growth stages.

### 2.4. Calibration and Validation of Satellite Data

#### 2.4.1. Satellite Data Calibration

Satellite imagery was calibrated using the mean ratio and temperature deviation methods to improve consistency with UAV data. The calibrated data were then used as inputs to the Random Forest (RF) model for PMC estimation across four maize growth stages: BBCH 11, BBCH 16, BBCH 61, and BBCH 65.

**Figure 6:**
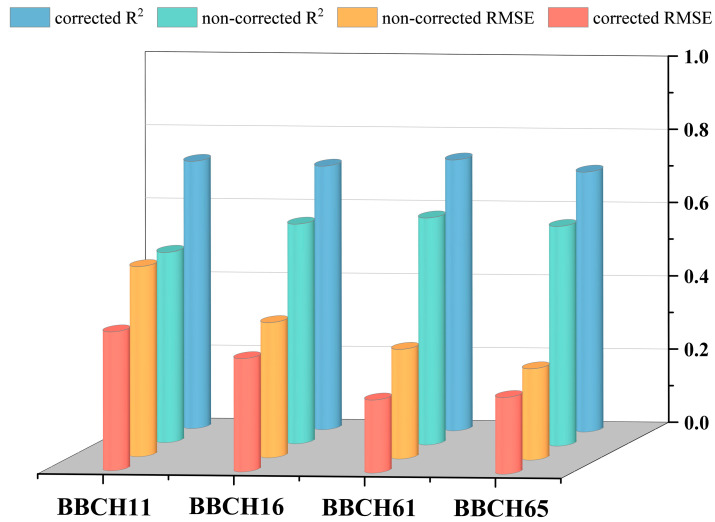
Comparison of PMC estimation accuracy of the RF model before and after satellite image calibration.

Model performance based on calibrated imagery was consistently higher than that based on uncalibrated data (Figure 6). For the calibrated models, R^2^ values reached 0.73, 0.72, 0.74, and 0.71 for the four stages, respectively, compared with 0.52–0.62 for the uncalibrated models. This corresponds to an average increase of approximately 0.12 in R^2^, indicating improved explanatory capability. A similar pattern was observed for RMSE. Prediction errors were reduced to 0.38%, 0.31%, 0.20%, and 0.21% after calibration, whereas higher values (0.25–0.52%) were obtained without calibration. On average, RMSE decreased by approximately 0.09%, reflecting improved prediction accuracy. BBCH 61 showed the most pronounced improvement, with R^2^ increasing from 0.62 to 0.74 and RMSE decreasing from 0.30% to 0.20%. This indicates that calibration plays a more critical role at this stage, likely due to stronger atmospheric and thermal influences on satellite observations. Across all growth stages, calibration enhanced model stability and accuracy, supporting the use of RF with calibrated satellite imagery for large-scale PMC estimation.

#### 2.4.2. Validation of Satellite Data

**Figure 7:**
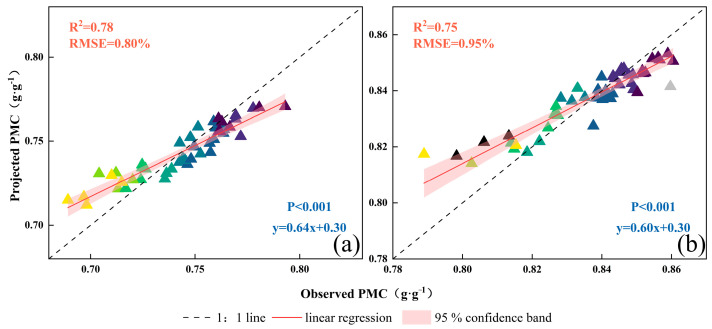
Validation of estimated plant moisture content against measured values in regions B (**a**) and C (**b**).

After satellite image calibration, the selected optimal feature variables were used as inputs for the RF model to estimate plant moisture content. The relationship between predicted and measured PMC in validation areas B and C was evaluated using scatter plots (Figure 7). The results show that the coefficients of determination (R^2^) reached 0.78 and 0.75 for regions B and C, respectively, indicating good agreement between predicted and observed values. The model maintained stable performance across different validation regions, suggesting satisfactory spatial transferability. In addition, consistent accuracy across regions implies that the model is less sensitive to regional variability. These findings indicate that the RF model based on calibrated satellite imagery can provide reliable estimates of PMC and has potential for large-scale monitoring in silage maize cultivation areas.

### 2.5. Spatial Distribution of Plant Moisture Content

#### 2.5.1. UAV-Based Spatial Inversion Distribution

**Figure 8:**
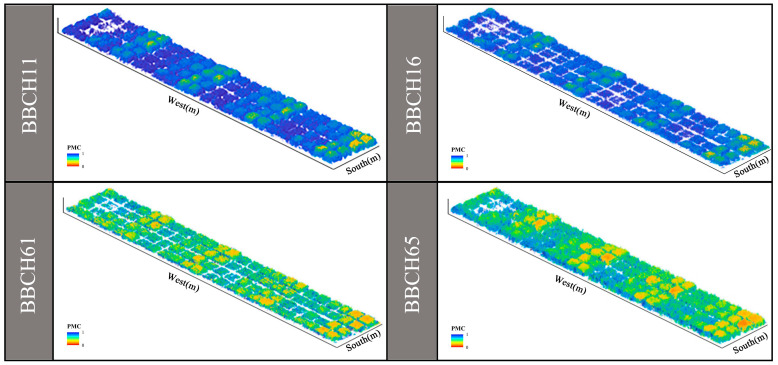
Spatial distribution of plant moisture content derived from UAV imagery.

The optimal RF model was applied to map the spatial distribution of PMC in silage maize across different growth stages. A decreasing trend in PMC was observed over the growing season, reflecting increased water consumption and physiological changes during crop development. As plants mature, internal water content declines, resulting in lower PMC values. The spatial distribution map (Figure 8) reveals clear heterogeneity within the study field. Higher PMC values were generally observed in the central areas, while lower values occurred toward the field edges. This spatial pattern is likely associated with variations in soil moisture conditions and field microenvironments. Central areas tend to retain more stable soil moisture, supporting higher plant water content. In contrast, edge zones are more susceptible to environmental influences such as wind and evaporation, which can reduce soil water availability and consequently decrease PMC. In addition, differences in irrigation and field management practices may further contribute to the uneven distribution of PMC across the field.

#### 2.5.2. Satellite-Based Spatial Inversion Distribution

**Figure 9:**
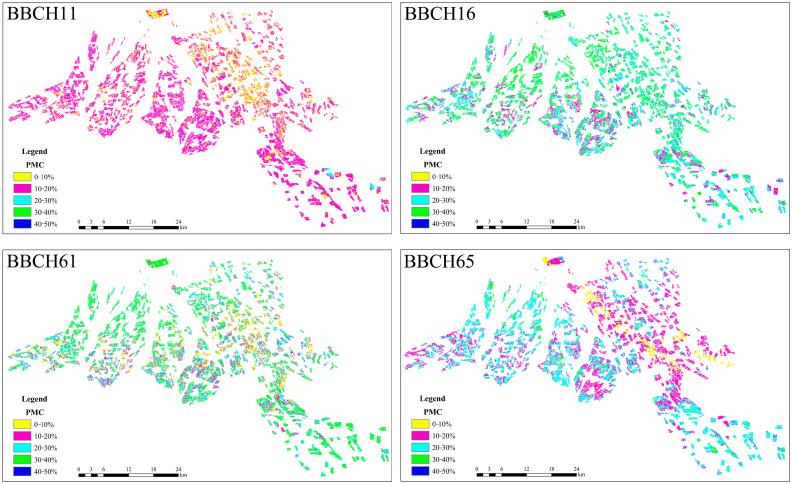
Spatial distribution map of plant moisture content inversion based on calibrated satellite imagery.

Satellite imagery calibrated using the mean ratio and temperature bias methods was combined with the RF model to estimate the spatial distribution of PMC across the study area. The temporal dynamics over four key growth stages—BBCH 11, BBCH 16, BBCH 61, and BBCH 65—are presented in Figure 9.

PMC exhibited a general pattern of increasing during early growth stages followed by a decline at later stages. At BBCH 11, PMC remained relatively low, with most areas falling within the 10–20% range, indicating limited water uptake capacity at early development. At BBCH 16, PMC increased markedly, accompanied by an expansion of medium- to high-value regions, reflecting enhanced water demand and uptake during rapid vegetative growth. PMC reached relatively high levels at BBCH 61, with a more homogeneous spatial distribution compared to earlier stages, although localized variability began to appear. By BBCH 65, PMC declined, with low-value areas expanding and high-value regions decreasing, consistent with physiological maturation and water redistribution within the plant.

Spatially, all stages exhibited a patchy distribution pattern, indicating notable heterogeneity within the field. These variations are likely associated with differences in soil moisture conditions, irrigation practices, and field management. Areas with higher PMC were generally linked to better water availability, whereas lower-PMC regions were more affected by water limitation and environmental factors. These results indicate that satellite-based PMC estimation can effectively capture both temporal dynamics and spatial variability, supporting its application in precision irrigation and water management.

## 3. Discussion

### 3.1. Role of Feature Selection in Model Performance Improvement

In remote sensing modeling and inversion studies, feature selection is a crucial step for improving model performance. It effectively removes redundant variables, reduces multicollinearity, and enhances model stability and generalization ability [15]. By selecting features that are highly correlated with the target variable, the model can better capture key information while reducing the risk of overfitting [16]. Among various methods, Pearson correlation analysis is widely used in spectral variable selection due to its simplicity and intuitive interpretability. Numerous studies have demonstrated that selecting spectral indices highly correlated with vegetation physiological parameters using Pearson correlation coefficients can significantly improve inversion model accuracy [17]. For example, F. Toscano et al. proposed a predictive correlation screening method for high-dimensional data analysis based on Pearson correlation, which effectively reduced prediction errors and improved model stability [18]. Similarly, K. Shackel systematically evaluated more than 30 hyperspectral vegetation indices and their relationships with crop growth parameters, confirming the effectiveness of this method in dimensionality reduction [19]. G. Vida et al. further applied Pearson correlation analysis to identify spectral bands and vegetation indices closely related to leaf chemical properties, significantly improving the efficiency of high-throughput spectral applications in crop monitoring [20]. These studies highlight that Pearson correlation analysis is a reliable and effective tool for feature selection in hyperspectral data processing [21].

In this study, Pearson correlation analysis was applied to systematically screen the extracted spectral indices, aiming to identify key indicators highly correlated with maize PMC. By calculating the correlation coefficients between each spectral index and the measured plant moisture content, variables with strong explanatory power and significant correlations were retained, while redundant or weakly correlated features were eliminated [22]. This process effectively simplified the model input variables and mitigated the impact of multicollinearity. The selected spectral indices were then used as key input features for model construction, significantly improving the fitting performance and prediction accuracy of the inversion models [23]. Moreover, feature selection enhanced model robustness and its potential for generalization, providing strong data support for accurate and efficient estimation of maize plant moisture content.

Based on the specific data obtained in this study, the feature variables selected through Pearson correlation analysis exhibited pronounced stage-dependent sensitivities. Among spectral indices, NDVI at BBCH 61 showed the strongest correlation with PMC (r = 0.87, *p* < 0.001), while NGRDI exhibited the strongest negative correlation at the same stage (r = −0.93, *p* < 0.001), indicating that canopy greenness and chlorophyll content are most sensitive to water status changes during this period. BBCH 16 and BBCH 65 demonstrated an increased contribution of textural features: TTI7 reached a correlation coefficient of −0.711 with PMC at BBCH 16, and TTI1 reached −0.845 at BBCH 65, suggesting that as canopy structure becomes more complex and leaf overlap increases, spectral information alone is insufficient to fully characterize water status, making spatial textural information a critical complement. Among temperature-related features, CWSI1 exhibited extremely strong negative correlations with PMC at both BBCH 61 (r = −0.95) and BBCH 65 (r = −0.99), confirming that thermal infrared indicators are highly sensitive to water stress during reproductive growth. This stage-dependent feature sensitivity analysis, grounded in our empirical data, demonstrates that no single type of remote sensing feature maintains optimal performance across all growth stages; rather, the dynamic combination of multi-dimensional features is key to improving PMC estimation accuracy. This finding further validates the necessity of our stage-specific feature selection strategy.

### 3.2. Impact of Satellite Data Calibration on Accuracy Improvement

When using satellite data to invert maize plant moisture content, low accuracy is often a major challenge. This is primarily due to the relatively coarse spatial resolution of satellite imagery, which limits its ability to capture subtle crop growth variations and spatial heterogeneity in plant conditions [24]. In addition, differences in spectral response and atmospheric effects can introduce biases into the inversion results. Although satellite data offer clear advantages for large-scale monitoring, these limitations restrict their effectiveness at the field scale [25].

To address the trade-off between the high accuracy but limited coverage of UAV data and the wide coverage but lower precision of satellite data, this study proposed a band calibration approach that integrates both data sources [26]. By comparing and calibrating satellite data with high-resolution UAV imagery, the spectral response and spatial representation of satellite data were effectively optimized, leading to a significant improvement in inversion accuracy [27]. Previous studies support this approach. For instance, Zhang et al. used high-precision UAV imagery as an intermediate scale to calibrate spectral discrepancies in satellite data, improving spatial consistency and spectral reliability in remote sensing inversion of coastal saline soils [28]. Similarly, Yang et al. performed dual calibration of satellite imagery using UAV data in both spatial and spectral domains, reducing systematic errors caused by scale differences and enhancing model generalization capability [27]. This integrated method overcomes the limitations of relying on a single data source and provides a novel pathway for large-scale precision agricultural monitoring. After calibration, satellite data not only retain their advantage of wide-area coverage but also achieve accuracy levels closer to UAV data [29]. This improvement provides robust data support for applications such as large-scale crop monitoring, precision fertilization, and crop growth assessment, ultimately promoting the development of precision agriculture toward greater scalability and efficiency [30].

Based on the calibration results of this study, the improvement in estimation accuracy following satellite data calibration was substantial. Prior to calibration, the RF model based on original Sentinel-2/Sentinel-3 imagery yielded R^2^ values of only 0.52–0.62 across growth stages, which increased to 0.71–0.74 after calibration—an average improvement of approximately 0.12. Concurrently, RMSE decreased from 0.25–0.52% before calibration to 0.20–0.38% after calibration, representing an average reduction of about 0.09%. The most pronounced improvement occurred at BBCH 61, where R^2^ increased from 0.62 to 0.74 (a 19.4% improvement) and RMSE decreased from 0.30% to 0.20% (a 33.3% reduction). This can be attributed to the higher atmospheric water vapor content and greater atmospheric interference on thermal infrared signals during this period, making the temperature deviation correction particularly effective. These results demonstrate that even though the thermal infrared spatial resolution of Sentinel-3 (approximately 1 km) is considerably coarser than that of UAV data, the response relationship with PMC can still be effectively recovered after cross-scale calibration using the mean ratio and temperature deviation methods. More importantly, the calibrated model achieved R^2^ values of 0.78 and 0.75 in independent validation zones B and C, respectively, confirming the stability and generalizability of the calibration approach. This quantitative analysis, grounded in our empirical data, indicates that cross-scale calibration is a critical technical step for achieving “knowledge transfer” from high-precision UAV data to wide-coverage satellite data, providing a reliable accuracy guarantee for regional-scale PMC estimation.

### 3.3. Effects of Different Inversion Models on the Estimation Accuracy of Maize Plant Moisture Content

Different machine learning models show notable differences in performance when applied to maize PMC inversion using remote sensing data [31]. Partial Least Squares Regression demonstrates strong capability in extracting complex feature relationships and achieves high prediction accuracy, especially when dealing with nonlinear interactions in multi-source remote sensing data. However, it generally requires larger datasets and higher computational resources [32]. The Random Forest model exhibits strong robustness and interpretability [33]. It can effectively handle high-dimensional features and evaluate variable importance, making it a reliable and stable choice for PMC estimation. Nevertheless, its ability to capture deep and complex feature relationships is slightly weaker compared to PLSR [34]. Support Vector Machine performs reasonably well under small-sample conditions, but its capability in handling high-dimensional remote sensing data is relatively limited [35]. Due to constraints associated with kernel functions, it is less effective in modeling complex feature spaces, which reduces its performance in intricate inversion tasks.

Under the data conditions of this study, the three models exhibited a clear performance hierarchy for PMC estimation in silage maize. The Random Forest (RF) model achieved the best results across all growth stages, with validation R^2^ ranging from 0.84 to 0.92 and RMSE from 0.10% to 0.50%; Support Vector Machine (SVM) ranked second with an R^2^ of 0.80–0.91; and Partial Least Squares Regression (PLSR) showed relatively lower performance with an R^2^ of 0.78–0.88. Taking BBCH 61, which yielded the highest accuracy, as an example, RF achieved an R^2^ of 0.92, surpassing SVM (0.91) and PLSR (0.88) by 0.01 and 0.04, respectively, with an RMSE of 0.10%, which was 0.05% and 0.08% lower than those of SVM (0.15%) and PLSR (0.18%). Although the absolute differences appear modest, RF consistently maintained the lowest RMSE and highest R^2^ across all four growth stages, demonstrating the best overall consistency. Further analysis of the built-in variable importance ranking in the RF model revealed that the top contributors to PMC at BBCH 61 were CWSI1, NDVI, and TTI1, in that order—corresponding precisely to the three physiological characteristics of this period: intensifying water stress, changes in canopy greenness, and increasing structural complexity. In contrast, SVM and PLSR were less effective at integrating feature information across different scales and types, particularly in the combined utilization of textural and thermal features, leading to significantly higher prediction errors at BBCH 16 and BBCH 65 compared to RF. This evidence, grounded in our specific model performance metrics and variable importance analysis, demonstrates that RF, as an ensemble learning method, has distinct advantages in handling multi-source heterogeneous remote sensing features, capturing nonlinear relationships, and resisting overfitting, making it a preferred modeling approach for multi-source remote sensing data fusion scenarios.

Research shows that the differences in model performance are mainly attributed to their algorithmic characteristics and adaptability to remote sensing data [36,37]. PLSR benefits from its ability to model latent variables and capture multi-level relationships, giving it an advantage in image-based analysis [38]. RF maintains balanced performance through ensemble learning and feature selection mechanisms [39], while SVM tends to underperform in complex scenarios [40]. In practical applications, it is essential to balance model complexity, computational cost, and accuracy requirements based on data characteristics [41].

### 3.4. Limitations and Future Research Directions

This study innovatively integrates UAV and satellite remote sensing data, combining band calibration and feature selection to construct a high-precision model for maize PMC inversion. This method demonstrated good application potential within the study region, providing a feasible technical reference for regional-scale moisture monitoring of silage maize in arid areas; however, its generalizability across different years and cultivars requires further validation. However, model performance is subject to site-specific environmental constraints and stage-dependent physiological dynamics. Specifically, atmospheric scattering and water vapor absorption—particularly for the Sentinel-3 thermal infrared bands (∼1 km resolution)—can significantly attenuate thermal signals, as reflected in the marked accuracy improvement following calibration at the tasseling stage (R^2^ increased from 0.62 to 0.74, a 19.4% gain). Soil background noise is another major source of uncertainty, especially at BBCH 11 and BBCH 16 when fractional vegetation cover is low; under such conditions, the reflectance signal captured by sensors contains a substantial soil component, which dilutes the sensitivity of spectral indices (e.g., NDVI: r = 0.53 at BBCH 11 versus r = 0.87 at BBCH 61). Field micro-environmental heterogeneity, including edge effects associated with wind erosion and enhanced evaporation, further contributes to within-field PMC variability, as shown in the UAV-derived spatial distribution maps (Figure 8), where edge zones consistently exhibit lower PMC than central areas. In addition, the current limitation in spectral coverage restricts the direct use of more physically based water-sensitive indices. The absence of shortwave infrared information in the available UAV multispectral data limits the incorporation of indices that directly respond to leaf water absorption features. We acknowledge that the availability of hyperspectral data or multispectral sensors equipped with SWIR bands in future studies would enable the inclusion of these water-sensitive indices, thereby further improving the physical interpretability of the model. Furthermore, we acknowledge that the random train–test split does not explicitly account for spatial dependence among samples, which may lead to a slight optimistic bias in performance evaluation. This limitation has been noted in the revised manuscript, and spatially explicit validation strategies will be considered in future work. Future research should focus on improving model generalization and applicability by incorporating multi-source data fusion techniques and developing growth stage-adaptive modeling strategies. These advancements would further enhance the robustness and practical value of PMC inversion models in precision agriculture.

## 4. Materials and Methods

### 4.1. Study Area Overview

**Figure 10:**
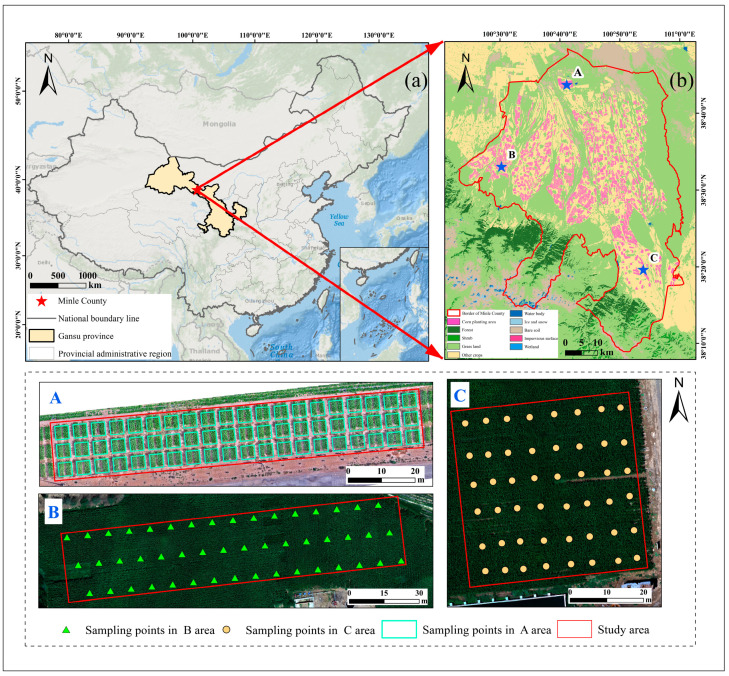
(**a**) The national location of the study area; (**b**) the location of Minle County in the study area; (**A**,**B**,**C**) the spatial distribution of the experimental plots of A, B and C, respectively.

**Figure 11:**
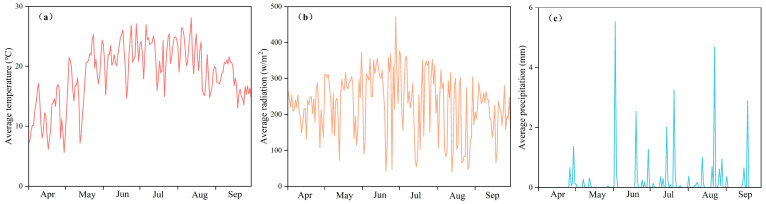
Daily meteorological conditions during the 2025 silage maize growing season: (**a**) temperature; (**b**) radiation; (**c**) precipitation.

The experiment was carried out at Huarui Ranch in Minle County, Zhangye City, Gansu Province, China (100°22′–101°13′ E, 37°56′–38°48′ N). The location of the study area, along with the distribution of sampling points, is presented in Figure 10. Situated within the Heihe River Basin, the region is characterized by a temperate continental desert steppe climate [14]. This area is marked by prolonged sunshine duration, pronounced diurnal temperature variation, and generally low precipitation. The mean annual temperature is around 6.5 °C. During summer, temperatures in July typically range from 25 to 27.9 °C, while winter conditions can be severe, with minimum temperatures in January dropping to between −18.9 and −21.39 °C. The frost-free period extends for approximately 140 days [42]. Annual average precipitation is relatively limited, totaling about 383.9 mm, whereas evaporation is substantially higher, ranging from 8686 to 9332 mm. The long-term average annual sunshine duration reaches approximately 2794 h [43]. Climatic conditions during the silage maize growing season are further illustrated in Figure 11. To improve water use efficiency, both drip irrigation and sprinkler irrigation are adopted in the region, with drip irrigation serving as the dominant method for silage maize cultivation.

### 4.2. Experimental Design and Plant Sample Preprocessing

#### 4.2.1. Experimental Design

The study area was classified into one main experimental zone (Zone A) and two supplementary validation zones (Zones B and C) according to local environmental conditions and maize growth characteristics (Figure 1). These zones were selected to support systematic measurements of plant moisture content. Sampling locations were determined based on two criteria. First, points were positioned near the center of observation areas to ensure strong spatial representativeness and homogeneous land-use conditions. Second, all sampling plots were located in maize fields with comparable growth stages and relatively uniform canopy conditions, ensuring consistency in the remote sensing signals captured across different sampling points.

**Figure 12:**
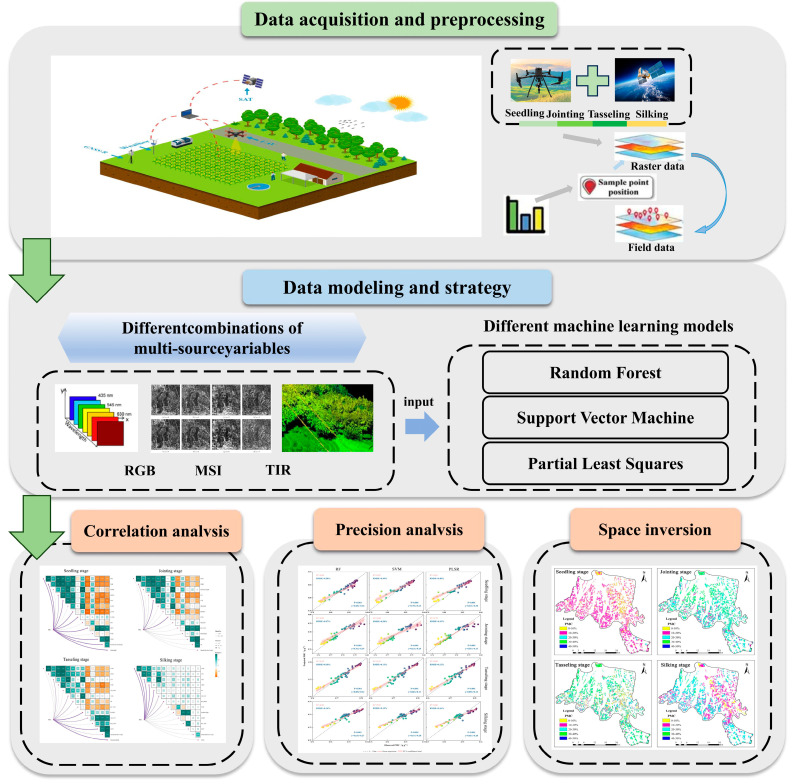
Overall workflow of the study.

The experiment was conducted between April and October 2025. The study area features predominantly sandy soils, relatively flat terrain, and is located close to drip irrigation infrastructure. A cultivation pattern combining full plastic film mulching with a double-ridge and furrow system was employed. Planting density was maintained at 6.75 × 104 plants ha^−1^, and drip irrigation was applied throughout the growing season, representing a typical intensive cultivation practice in the region. The maize cultivar used in this study was “Jinling 67.” Crops were planted with a row spacing of 50 cm and a plant spacing of 25 cm. The overall workflow is shown in Figure 12.

#### 4.2.2. Measurement of Plant Moisture Content

A total of 60 sampling sites were evenly distributed in the core experimental area (Zone A), and 48 sites each in the validation areas (Zones B and C), with all geographic coordinates recorded using GPS. At each sampling site, all maize plants within a 5 cm radius of the central coordinate point were cut at the stem base (at ground level) to collect the entire aboveground portion, including stems, leaves, and ears (if present). From the harvested pool, three representative plants with uniform growth vigor were selected and combined to form a single composite sample per site. The composite sample was immediately placed into a fresh-keeping bag, and its total fresh weight (FW) was recorded using an electronic balance (accuracy 0.01 g); the individual sample fresh weight typically ranged from 500 to 800 g, varying with growth stage.

In the core experimental area (Zone A), samples were collected on 10 May, 15 June, 13 July, and 18 August 2025. In the validation areas, samples were collected on 5 June and 21 July 2025 (Zone B), and on 15 June and 18 July 2025 (Zone C). After fresh weight determination, all samples were oven-dried at 105 °C for 30 min to inactivate enzymes, followed by continuous drying at 70 °C for approximately 48 h until constant weight was achieved to determine the dry weight (DW) [44]. The plant moisture content (PMC) was calculated using the wet-basis moisture content formula as follows:

(1) $$\mathrm{PMC}=\frac{\mathrm{FW}\;-\;\mathrm{DW}}{\mathrm{FW}}\times 100\%$$

where PMC represents plant moisture content on a wet basis (%); FW denotes the fresh weight of the plant (mg); and DW denotes the dry weight of the plant (mg).

### 4.3. Remote Sensing Data Acquisition and Processing

#### 4.3.1. UAV Data Acquisition and Processing

In this study, UAV-based remote sensing data were acquired using a DJI M300 RTK (Shenzhen China) platform equipped with an MS600 Pro multispectral sensor and a Zenmuse H20T module, enabling the collection of multispectral, LiDAR point cloud, and thermal infrared data. In addition, a DJI Matrice 4E UAV (Shenzhen China) was deployed to obtain high-resolution visible imagery. The MS600 Pro sensor (Qingdao China) captures six spectral channels, including blue (450 nm @ 35 nm), green (555 nm @ 27 nm), red (660 nm @ 22 nm), red edge 1 (720 nm @ 10 nm), red edge 2 (750 nm @ 10 nm), and near-infrared (840 nm @ 30 nm). With a flight altitude of 30 m, the acquired multispectral imagery achieved a ground sampling distance (GSD) of 4.09 cm. The Zenmuse H20T (Shenzhen China) incorporates an uncooled vanadium oxide (VOx) thermal sensor, with a temperature detection range from −40 °C to 550 °C. The DJI Matrice 4E is equipped with a multi-camera imaging system, consisting of a wide-angle camera (4/3-inch CMOS, 20 MP, f/2.8–f/11, 24 mm equivalent), a medium telephoto camera (1/1.3-inch CMOS, 48 MP, f/2.8, 70 mm equivalent), and a telephoto camera (1/1.5-inch CMOS, 48 MP, f/2.8, 168 mm equivalent).

UAV data were collected under clear sky conditions between 10:00 and 12:00 local time using a nadir imaging configuration with equal-interval image acquisition. Flight missions were planned in advance over the study area, with a flight altitude of 30 m, a speed of 2.5 m s^−1^, and a ground sampling distance (GSD) of 4.09 cm. The forward and side image overlaps were set to 80% and 75%, respectively. Four ground control panels (40 × 40 cm) were installed at the corners of the field and maintained throughout the growing season to serve as ground control points (GCPs). Their coordinates were measured using real-time kinematic (RTK) positioning. In addition, a reflectance calibration panel with known properties was used to support radiometric correction of the multispectral imagery. Image processing was carried out in Pix4DMapper (version 4.8.0, Pix4D S.A., Prilly, Switzerland), where mosaicking and radiometric correction were applied to produce high-resolution orthomosaic datasets (visible, multispectral, and thermal infrared), along with LiDAR point cloud data in LAS format. The resulting imagery was further processed in ENVI 5.6 to remove soil background using a maximum likelihood classification approach, yielding refined UAV datasets for subsequent analysis [45].

#### 4.3.2. Satellite Data Acquisition and Processing

Satellite-based visible, multispectral, and thermal infrared data were sourced from the European Space Agency (ESA) Copernicus Open Access Hub (https://dataspace.copernicus.eu/, accessed on 27 June 2026) [46]. Visible and multispectral imagery was obtained from the Sentinel-2 constellation (Sentinel-2A and Sentinel-2B), while thermal infrared data were derived from Sentinel-3. Sentinel-2 provides high-resolution optical imagery, with a spatial resolution of 10 m for visible bands. Its onboard Multispectral Instrument (MSI) acquires data across 13 spectral bands (440–2200 nm) at spatial resolutions of 10 m, 20 m, and 60 m, covering the visible, red-edge, near-infrared, and shortwave infrared regions. Sentinel-3 carries the Sea and Land Surface Temperature Radiometer (SLSTR), which records thermal infrared data across nine spectral channels, with spatial resolutions ranging from 300 m to 1.2 km. With a revisit interval of approximately 5–6 days, these datasets provide consistent temporal coverage for monitoring the study area. Preprocessing, including atmospheric correction and resampling, was conducted using the Sentinel Application Platform (SNAP). The processed imagery was then imported into ENVI 5.6 for subsequent analysis, including mosaicking, reflectance extraction, image clipping, inversion, classification, and final product generation [47].

#### 4.3.3. Calibration of UAV and Satellite Data

To reduce inconsistencies between UAV observations in the test area (Zone A) and satellite data covering the broader study region, a cross-scale calibration was applied. Reflectance and canopy temperature values at each sampling location were extracted from both UAV and satellite datasets for comparison. For visible and multispectral data, calibration was implemented using the mean ratio approach to derive a reflectance correction coefficient (C) [48]. This method improves consistency between data sources and enhances the reliability of subsequent analyses:

(2) $$C=\frac{\sum_{i=1}^{n}B_{\mathrm{UAV}}/B_{\mathrm{Sentinel}}}{n}$$

For thermal infrared data, a temperature deviation approach was adopted for calibration. The mean canopy temperature difference (${∆T}_{\mathrm{mean}}$) at the sampling locations was first estimated and subsequently used to adjust the satellite-derived thermal imagery across the study area. The corresponding formulation is given as follows:

(3) $$∆T_{n}\;=\;T_{\mathrm{uav}\_\mathrm{mean}\_n}\;-\;T_{\mathrm{sat}\_\mathrm{mean}\_n}$$

(4) $${∆T}_{\mathrm{mean}}=\mathrm{mean}({∆T}_{1},{\;∆T}_{2},\ldots ,{\;∆T}_{n})$$

(5) $$T_{\mathrm{sat}\_\_\mathrm{corrected}(x,y)\;}=T_{\mathrm{sat}\_\_\mathrm{original}(x,y)\;}+{\;∆T}_{\mathrm{mean}}$$

### 4.4. Feature Extraction

Feature selection based on correlation analysis helps reduce data redundancy, improve model robustness, and enhance computational efficiency by identifying the most informative variables and removing highly correlated or irrelevant features. Based on UAV and satellite observations, three types of features were extracted, including vegetation indices (VIs), temperature-related variables (TCs), and texture metrics (TTIs). Vegetation indices and texture features are effective in separating vegetation from soil background and provide important information on crop growth status, which is closely linked to plant moisture content. Temperature-related features describe the spatial consistency of crop canopies. Higher uniformity in canopy temperature is generally associated with more favorable growth conditions, making these variables particularly useful for PMC estimation [49].

#### 4.4.1. Vegetation Index Extraction

Variations in irrigation regimes can lead to noticeable differences in vegetation spectral responses, particularly in terms of absorption and reflectance across multiple bands. Vegetation indices are derived from reflectance values at specific wavelengths and are widely used to enhance vegetation-related signals while reducing background interference [50]. In this study, spectral reflectance obtained from UAV-based multispectral imagery was used to compute a set of vegetation indices. The selection of indices was based on spectral availability and their established applicability in crop monitoring studies. Accordingly, 10 commonly used vegetation indices were selected for analysis (Table 2).

#### 4.4.2. Texture Index Extraction

To explore the usefulness of texture information from UAV multispectral imagery in characterizing the water status of silage maize, texture features were extracted using the gray-level co-occurrence matrix (GLCM). The GLCM describes the spatial distribution of grayscale values by quantifying how often pairs of pixel intensities occur under defined spatial configurations. Through this representation, structural patterns in the image, such as heterogeneity and spatial arrangement, can be effectively captured. Compared with more complex approaches, GLCM-based analysis relies on gray-level statistics and does not involve iterative optimization, making it computationally efficient and suitable for processing large datasets. Based on this method, nine texture metrics were derived, including angular second moment, contrast, dissimilarity, energy, entropy, homogeneity, maximum probability, mean, and standard deviation [60]. All texture features were calculated within a Python 3.12.12 environment.

Given the large number of texture metrics, this study further constructed eight texture indices (TTIs) to evaluate the feasibility of different combinations of texture measures for diagnosing plant water status. In the formulas, $T_{\lambda 1}$, $T_{\lambda 2}$ and $T_{\lambda 3}$ represent combinations of any three texture measures [61]. By calculating and analyzing all possible combinations, the optimal set was selected and used as input variables for model development. The calculation formula is as follows:

(6) $$TT1=\frac{T_{\lambda 1}}{T_{\lambda 2}\times T_{\lambda 3}}$$

(7) $$TT2=\frac{T_{\lambda 1}}{T_{\lambda 2}+T_{\lambda 3}}$$

(8) $$TT3=\frac{T_{\lambda 1}-T_{\lambda 2}}{T_{\lambda 2}+T_{\lambda 3}}$$

(9) $$TT4=\frac{T_{\lambda 1}-T_{\lambda 2}}{T_{\lambda 2}-T_{\lambda 3}}$$

(10) $$TT5=\frac{T_{\lambda 2}\times T_{\lambda 3}}{T_{\lambda 1}}$$

(11) $$TT6=\frac{T_{\lambda 2}+T_{\lambda 3}}{T_{\lambda 1}}$$

(12) $$TT7=(T_{\lambda 1}-T_{\lambda 2})-(T_{\lambda 2}-T_{\lambda 3})$$

(13) $$TT8=\frac{T_{\lambda 1}-T_{\lambda 2}}{\left[(T_{\lambda 1}-T_{\lambda 2})-(T_{\lambda 2}-T_{\lambda 3})\right]}$$

#### 4.4.3. Temperature Feature Extraction

(1) Canopy temperature extraction.

To evaluate the effectiveness of canopy temperature derived from thermal infrared imagery, the Otsu thresholding algorithm was applied to extract silage maize canopy pixels. Based on the segmented canopy pixels, canopy temperature within the experimental area was calculated.

(2) Computation of canopy thermal indices.

The Crop Water Stress Index (CWSI) and the relative stomatal conductance index (Ig) were calculated using canopy temperature derived from UAV thermal infrared imagery, along with the lower limit ($T_{wet}$) and upper limit ($T_{dry}$) of canopy temperature under the same meteorological conditions [62]. These thermal indices, constructed based on canopy temperature, have been widely recommended for diagnosing crop water status and are extensively documented in the literature [49]. The specific calculation formulas are as follows:

(14) $$CWSI1=\frac{\left(T_{dry}-T_{c}\right)}{T_{dry}}$$

(15) $$CWSI2=\frac{\left(T_{dry}-T_{c}\right)}{\left(T_{dry}-T_{wet}\right)}$$

(16) $$CWSI3=\frac{\left(T_{c}-T_{wet}\right)}{T_{wet}}$$

(17) $$CWSI4=\frac{\left(T_{c}-T_{wet}\right)}{\left(T_{dry}-T_{wet}\right)}$$

(18) $$Ig=\frac{\left(T_{dry}-T_{c}\right)}{\left(T_{c}-T_{wet}\right)}$$

where $T_{c}$, $T_{\mathrm{wet}}$ and $T_{\mathrm{dry}}$ represent canopy temperature, leaf temperature under well-watered conditions (lower limit of canopy temperature), and leaf temperature under extreme water stress conditions (upper limit of canopy temperature), respectively, all expressed in °C.

### 4.5. Model Construction and Validation

A plant moisture content estimation model was developed using an optimal combination of vegetation indices, texture features, and temperature-related variables derived from remote sensing data. Model performance was evaluated using three machine learning approaches: random forest (RF), back propagation neural network (BPNN), and support vector machine (SVM). Each field sample was spatially matched to the corresponding pixel in the remote sensing imagery. The dataset was randomly split into training and testing subsets at a ratio of 2:1. The selected features after screening were used as input variables, while plant moisture content served as the target variable.

The random forest (RF) model uses bootstrap sampling to generate multiple training subsets, while feature selection is performed randomly during tree construction. Based on preliminary experiments, the minimum number of samples per leaf node (min_leaf) and the number of trees (ntree) were set to 5 and 100, respectively [63].

Partial Least Squares Regression (PLSR) extracts latent components from both predictors and responses, maximizing their covariance while reducing dimensionality, which effectively addresses multicollinearity among variables. Prior to modeling, all input features and output variables were scaled to the [0, 1] range using Min-Max normalization to eliminate dimensional effects. The number of latent variables was set to 14, equal to the number of input features, making PLSR equivalent to ordinary least squares regression and thereby preserving all original information. The model parameters were solved using the built-in plsregress function in MATLAB, which adopts the default SIMPLS algorithm for matrix decomposition. As a linear regression method, it requires no iterative training, and thus no hyperparameters such as learning rate or iteration number are involved. The resulting regression coefficient matrix β was used to compute predicted values for both training and test sets, followed by reverse normalization to restore the original scale [64].

Support vector machine (SVM) was applied to establish the regression model by balancing model complexity and estimation error. During training, the model determines the optimal solution by maximizing the margin under given constraints, rather than relying on iterative weight updates. A radial basis function (RBF) kernel was selected, with the penalty parameter (C) and kernel parameter (γ) set to 0.8 and 4, respectively [65].

Model performance for PMC estimation was assessed using the coefficient of determination (R^2^) and root mean square error (RMSE) for both calibration and validation datasets across the RF, PLSR, and SVM models. These metrics were used to compare different models and determine the most appropriate approach. All analyses were conducted in MATLAB R2021b. R^2^ describes the proportion of variance explained by the model, whereas RMSE reflects the average deviation between predicted and observed values [66].

## 5. Conclusions

In this study, we established a regional-scale estimation framework for maize plant moisture content by integrating UAV and satellite remote sensing data through cross-scale calibration and multi-dimensional feature selection. The results confirm the feasibility and reliability of this framework for large-scale PMC characterization under arid agricultural conditions. The main conclusions are as follows:

(1) Among the evaluated models, the Random Forest model achieved the best performance, with R^2^ values ranging from 0.84 to 0.92, indicating strong predictive capability across different growth stages.
(2) Satellite data calibration significantly improved inversion accuracy, with R^2^ increasing from 0.52–0.62 to 0.71–0.74 after calibration, demonstrating the importance of reducing scale and sensor discrepancies.
(3) The integration of UAV and satellite data provides an effective approach for improving the accuracy of large-scale PMC estimation, enabling more reliable monitoring of crop water status.

## Figures and Tables

**Table 1:** Summary statistics of plant moisture content at different growth stages in 2025.

Growth Stage	Max	Min	Mean	SD	CV
BBCH 11	0.98	0.84	0.89	0.03	0.03
BBCH 16	0.90	0.61	0.82	0.05	0.06
BBCH 61	0.94	0.62	0.80	0.10	0.12
BBCH 65	0.89	0.55	0.74	0.07	0.09

**Table 2:** Vegetation indices derived from UAV-acquired multispectral reflectance.

Full Name	Computing Formula	References
Normalized Difference Vegetation Index (NDVI)	(Rnir − Rred)/(Rnir + Rred)	[51]
Normalized Difference RedEdge (NDRE)	(Rnir − Rrededge)/(Rnir + Rrededge)	[52]
Normalized Green Red Difference Index (NGRDI)	(Rgreen − Rred)/(Rgreen + Rred)	[53]
Green Normalized Difference Vegetation Index (GNDVI)	(Rnir − Rgreen)/(Rnir + Rgreen)	[50]
Blue Normalized Difference Vegetation Index (BNDVI)	(Rnir − Rblue)/(Rnir + Rblue)	[54]
Renormalized difference vegetation index (RDVI)	(Rnir − Rred)/(sqrt(Rnir + Rred))	[55]
Ratio Vegetation Index (RVI)	Rnir/Rred	[56]
Chlorophyll Index (CI)	Rnir/Rrededge − 1	[57]
Difference Vegetation Index (DVI)	Rnir − Rred	[58]
Transform vegetation index (TVI)	60 × (Rnir − Rgreen) − 100 × (Rred − Rgreen)	[59]

Note: Rblue, Rgreen, Rred, Rrededge, and Rnir denote the reflectance values of the blue, green, red, red-edge, and near-infrared bands.

## Data Availability

All data used during the study is proprietary or confidential and only limited data can be provided.
